# Potential application of mono-, dual-, and triple-target GLP-1 receptor agonists in improving the prognosis of patients with diabetic foot ulcers

**DOI:** 10.3389/fendo.2025.1754925

**Published:** 2026-01-21

**Authors:** Zhe Li, Xujing Wang, Yan He, Keyan Hu, Yanyun Liu, Weiguang Zhang, Yujin Ma, Hongwei Jiang

**Affiliations:** 1 Luoyang Key Laboratory of Clinical Multiomics and Translational Medicine, Henan Key Laboratory of Rare Diseases, Endocrinology and Metabolism Center, The First Affiliated Hospital, and College of Clinical Medicine of Henan University of Science and Technology, Luoyang, China; 2 Department of Pharmaceutical Sciences, School of Basic Medical Sciences, Henan University of Science and Technology, Luoyang, China; 3 People’s Hospital of Baofeng, Pingdingshan, China; 4 Yibin Vocational College of Medicine and Health, Yibin, China; 5 Senior Department of Nephrology, The First Medical Center of Chinese PLA General Hospital, Chinese PLA Institute of Nephrology, National Key Laboratory of Kidney Diseases, National Clinical Research Center for Kidney Diseases, Beijing Key Laboratory of Kidney Diseases Research, Beijing, China

**Keywords:** cardiovascular risk, diabetic foot ulcer, GLP-1 receptor agonist, individualized therapy, multi-target agonists

## Abstract

Diabetic foot ulcer (DFU), a severe chronic complication of diabetes mellitus, poses a major public health threat due to its high rates of disability, recurrence, and all-cause mortality. The mortality rate of DFU patients is closely related to cardiovascular events, indicating that their treatment should go beyond local wound management and focus on cardiovascular risk intervention. Glucagon-like peptide-1 receptor agonists (GLP-1 RA), known for their cardiovascular protective effects demonstrated in cardiovascular outcome trials, offer new treatment opportunities for DFU patients. However, the pharmacological properties of GLP-1 RA that suppress appetite and delay gastric emptying may exacerbate malnutrition in DFU patients during the acute infection phase, limiting their use. This review aims to systematically describe personalized application strategies for GLP-1 RA based on the clinical staging differences in DFU patients. Additionally, it compares the clinical translational potential of mono-, dual-, and triple-target GLP-1 RA drugs and evaluates their adverse effects on DFU patients, with the aim of providing more rational and structured treatment strategies for improving the long-term prognosis of DFU patients.

## 1 Introduction

Diabetic foot ulcer (DFU) is a common yet severe complication among diabetic patients, with a lifetime prevalence of approximately 19%–34% (1). The pathogenesis of DFU is complex, involving multiple factors such as neuropathy, vascular lesions, infections, bone destruction, and biomechanical abnormalities. Its diagnosis and treatment often require multidisciplinary collaboration, leading to a significant increase in medical burden (2). Although comprehensive treatment methods, including active infection control, local debridement, and lower limb vascular reconstruction, can effectively promote wound healing, clinical follow-up reveals that DFU patients face not only prolonged or recurrent wound healing issues but also extremely high mortality risks. Reportedly, the overall mortality rate for DFU is high, with a 5-year mortality rate for DFU exceeds 50% (3), reaching 70% in those undergoing major amputation (4).

This significantly elevated mortality rate is not solely attributed to local foot lesions but is closely linked to systemic cardiovascular events. A meta-analysis of 8602 diabetic patients found that the all-cause cardiovascular morbidity in DFU patients is approximately 37.1%, and compared to patients without DFU, those with DFU have a 2.5-fold increased risk of all-cause cardiovascular-related mortality (5). This suggests that, in addition to lower limb arterial disease, coronary atherosclerosis and cerebrovascular stenosis may be widespread in the DFU patient population, contributing to multi-organ vascular damage. The non-healing of DFU wounds is often seen as a visible manifestation, while the real crisis lurks in the systemic vascular network. Therefore, improving the prognosis of DFU patients requires moving beyond traditional wound healing approaches and incorporating cardiovascular risk management as a long-term treatment goal.

Currently, sodium-glucose co-transporter 2 inhibitors (SGLT2i) have been widely applied in diabetic patients due to their heart and kidney protective effects, with expert consensus recommending the use of SGLT2i (except for canagliflozin) in diabetic foot patients without ulcers (6). However, the application of GLP-1 RA, which also provides cardiovascular and renal benefits, in DFU patients remains relatively scarce. A recent large-scale retrospective study showed that compared with SGLT2i, the use of GLP-1 RA in type 2 diabetes patients resulted in a longer survival time without the need for major amputation (7), suggesting that GLP-1 RA may reduce the risk of major amputation in DFU patients. Based on this, this review aims to systematically compare the clinical translational potential of mono-, dual-, and triple-target GLP-1 RA drugs, deeply analyze their adverse effects on DFU patients, and explore personalized application strategies for GLP-1 RA in DFU patients to provide new perspectives for improving their long-term prognosis.

## 2 GLP-1 RA cardiovascular protection and DFU benefit mechanisms

Recent large cardiovascular outcome trials (CVOTs) have provided high-quality evidence supporting the clinical application of GLP-1 RA. The LEADER trial for liraglutide, the SUSTAIN-6 trial for semaglutide, and the REWIND trial for dulaglutide have all consistently shown that these drugs reduce the risk of major adverse cardiovascular events (8–10). These cardiovascular protective effects are not limited to glucose control but involve multiple mechanisms independent of glucose regulation. The cardiovascular protection mechanisms of GLP-1 RA are multifaceted, including improvement of endothelial function (through nitric oxide release), inhibition of inflammation and oxidative stress, enhancement of lipid metabolism, weight reduction, and direct effects on myocardial and vascular smooth muscle cells (11).

In DFU patients, GLP-1 RA may offer additional benefits by promoting angiogenesis, improving blood flow perfusion, inhibiting the progression of neuropathy, reducing systemic inflammation, and accelerating wound healing. Zhu et al. found that GLP-1 RA promotes the expression of vascular endothelial growth factor, enhancing angiogenesis and improving blood supply to ischemic tissues (12), which is crucial for DFU patients who commonly experience microcirculatory disorders and lower limb ischemia. Diabetic peripheral neuropathy (DPN) is a major risk factor for the occurrence and development of DFU. Animal studies have shown that GLP-1 RA can improve DPN by modulating oxidative stress, inflammation, and extracellular matrix remodeling (13). DFU patients are often in a chronic inflammatory state, which delays wound healing. Ma et al. suggested that GLP-1 RA can reduce the adhesion of inflammatory cells to endothelial cells and modulate endothelial cell inflammatory responses, thereby exerting anti-inflammatory effects (14). Furthermore, research has confirmed that the application of GLP-1 RA can significantly shorten the healing time of DFU ulcers in mice and improve healing quality (15). It is worth noting that the benefits in DFU populations are mainly based on indirect or transformed evidence rather than direct clinical data, and more clinical exploration is needed.

However, a key clinical paradox arises with the use of GLP-1 RA in DFU patients: their pharmacological properties may pose risks during the acute phase of DFU. GLP-1 RA suppress appetite and delay gastric emptying, which are beneficial in stable patients for weight management but may be problematic during the acute infection phase of DFU. During this phase, patients are often in a high catabolic state, requiring adequate nutritional support to combat infection, maintain immune function, and promote wound healing. Adequate nutritional intake and the prevention of malnutrition are essential for effective wound healing, as poor nutrient intake and deficiencies have been linked to worse healing outcomes in DFU patients (16, 17). The appetite suppression caused by GLP-1 RA may not only lead to inadequate nutritional intake but also result in nausea, vomiting, and early satiety, consistent with well−documented gastrointestinal adverse events of GLP-1 RA (18, 19). These gastrointestinal effects, such as delayed gastric emptying, could further impair food intake and disrupt nutritional status, although studies specifically focused on acute DFU populations are limited. Therefore, the use of GLP-1 RA during the acute infection phase of DFU is not advisable, and glucose control strategies that ensure adequate nutritional intake and minimize gastrointestinal side effects should be prioritized.

## 3 Comparison of different GLP-1 RA drugs

### 3.1 Monotarget agonists

Monotarget GLP-1 RA (such as liraglutide, semaglutide, dulaglutide) act by mimicking incretin hormones. Their mechanisms include glucose-dependent insulin secretion, delayed gastric emptying, and enhanced satiety (20). Besides their metabolic advantages, such as glucose control and weight loss, these drugs have been validated in long-term, large-scale cardiovascular outcome trials, showing clear cardiovascular benefits (8–10). For DFU patients, the cardiovascular protective effect of these drugs is crucial, and *post-hoc* analyses have shown a reduction in the incidence of DFU-related adverse events (21).

### 3.2 Dual-target agonists

#### 3.2.1 GLP-1/GIP dual-target agonists

Tirzepatide is currently the most widely used GLP-1/Glucose-dependent insulinotropic polypeptide (GIP) dual-target agonist. GIP is another key incretin hormone predominantly expressed in adipose tissue, where it participates in glucose uptake, lipolysis, and regulation of lipoprotein lipase activity (22). When GLP-1 and GIP act synergistically, the appetite suppression and increased energy expenditure of GLP-1 counterbalance GIP’s tendency to promote fat accumulation, resulting in superior weight loss and metabolic improvement. A meta-analysis of 3484 patients confirmed that tirzepatide outperforms other drugs in terms of HbA1c control and weight loss (23). For DFU patients, the dual-target advantage provides significant weight reduction, reducing foot pressure and helping to prevent pressure ulcer recurrence. Moreover, improved blood glucose control further mitigates the effects of hyperglycemia on nerves and blood vessels, optimizing the wound healing microenvironment.

#### 3.2.2 GLP-1/GCG dual-target agonists

Glucagon (GCG) is generally considered a glu-elevating hormone, but its receptor activation also promotes energy expenditure and reduces liver fat (24). The GLORY-1 study of mazdutide demonstrated that it can induce weight loss in overweight or obese adults and reduce liver fat content and various cardiac metabolic risk factors (25). Recently, mazdutide has been approved by the National Medical Products Administration of China for blood glucose and weight control. Pemvidutide shows significant effects on metabolic dysfunction-related fatty liver disease (MALSD), with 24-week trial data showing a 50% reduction in liver fat content in 84.6% of participants (26). This has potential therapeutic implications for DFU patients with fatty liver, as fatty liver is a major driver of insulin resistance and systemic inflammation. Survodutide is undergoing a phase III cardiovascular safety study to evaluate its efficacy and safety in adults with BMI ≥ 27 kg/m² and cardiovascular disease or chronic kidney disease risk factors (27).

### 3.3 Triple-target and multitarget agonists

Retatrutide is a triple-target agonist that simultaneously activates GLP-1, GIP, and GCG receptors, achieving a “triple” metabolic effect—significant blood glucose reduction, substantial weight loss, and increased energy expenditure (28). Safety profiles indicate that Retatrutide’s adverse events are similar to those of GLP-1 and GLP-1/GIP receptor agonists, with gastrointestinal symptoms being the most common, typically mild to moderate (29). Notably, recent studies show that Retatrutide induces weight loss without a higher proportion of lean mass loss (30) and can reduce liver fat content in patients with MASLD by up to 86% (31). MWN105 is a GLP-1/GIP/FGF21 triple agonist. Fibroblast growth factor 21 (FGF21) improves blood glucose and lipid metabolism in diabetic mice (32). Several clinical studies have observed that in type 2 diabetes patients, especially women with carotid or lower limb arterial sclerosis, elevated serum FGF21 may be a compensatory mechanism against atherosclerosis (33, 34). Liskiewicz presented the GLP-1R/GIPR/Pan-peroxide enzymoid proliferation quintuple agonist at the 2025 European Association for the Study of Diabetes meeting, the latter activates three subtypes: α, δ, and γ, and has characteristics of enhancing insulin sensitivity and regulating dyslipidemia (35). The theoretical advantage of such multitarget agonists is to achieve more comprehensive and profound metabolic remodeling, potentially producing multiple positive effects on vascular lesions, neuropathy, and inflammation in DFU patients.

## 4 Adverse effects of GLP-1 RA in DFU patients

GLP-1 RA’s common adverse effects include gastrointestinal issues due to delayed gastric emptying and appetite suppression, particularly in the early stages of treatment (36). These side effects may diminish over time. DFU patients may be in a high metabolic stress state, significantly increasing their need for protein and energy. The gastrointestinal side effects caused by GLP-1 RA may lead to deterioration of the patient’s overall nutritional status, even exacerbating hypoproteinemia, anemia, and electrolyte disorders. Moreover, delayed gastric emptying may interfere with the absorption of oral medications such as antibiotics, circulatory drugs, or analgesics. Therefore, when initiating GLP-1 RA in DFU patients, it should start with a low dose, and gastrointestinal reactions and nutritional status should be closely monitored. Another concerning adverse effect is the decrease in muscle mass, with studies indicating that GLP-1 RA may be associated with some degree of muscle mass loss during weight loss, and the specific mechanisms are not yet fully unclear (37). DFU patients often have limited mobility and decreased muscle strength due to diabetic neuropathy, vascular lesions, pain, or amputation. A decrease in muscle mass may further impair the activity ability of DFU patients and increase the risk of osteoporosis. In addition, there is a double-edged sword effect in the cardiovascular system; some patients may experience tachycardia after using GLP-1 RA, and recent studies have reported that this may be related to the direct effect of GLP-1 RA on the sinoatrial node (38). Although GLP-1 RA has overall cardiovascular protective effects, persistent tachycardia may increase cardiac workload or mask other cardiovascular issues. Therefore, when applying GLP-1 RA in DFU patients, heart rate should be closely monitored, and the benefits and risks should be carefully weighed for patients with existing tachycardia or severe cardiovascular disease.

## 5 Personalized treatment recommendations for GLP-1 RA in DFU patients

Given that the risk-benefit ratio of GLP-1 RA varies dramatically in different clinical scenarios, we classify DFU patients into three clinical stages: acute infection phase, wound healing phase, and prevention phase, and then adopt corresponding GLP-1 RA application strategies.

Patients in the acute infection phase typically present with active foot infections (such as cellulitis, abscess, osteomyelitis) or severe ischemia (tissue necrosis). These patients often exhibit symptoms of infection, such as fever, elevated inflammatory markers, and local redness and swelling, with some also showing signs of lower limb ischemia, such as purple discoloration of the toes or absence of foot dorsalis pedis pulse. This phase is usually seen in the early hospitalization period, where treatment measures are particularly complex. Clinically, aggressive antibiotic therapy combined with necrotic tissue debridement is commonly used to rapidly control infection, restore tissue blood supply, and prevent the progression to amputation. In this phase, it is suggested that GLP-1 RA not be initiated, and that those already using it may need to suspend treatment. This decision is based on the fact that the primary therapeutic goals in this phase are “limb preservation and life preservation,” not long-term cardiovascular benefits, and that GLP-1 RA may exacerbate nutritional deterioration in patients, particularly with gastrointestinal side effects leading to insufficient nutrient intake and dehydration, which could severely impair the patient’s overall recovery and wound healing capacity.

The wound healing phase signifies that the patient’s condition has improved, the acute infection is well controlled, and the wound is in the healing process. Key features include the recovery of oral intake and gradual improvement in nutritional status. The clinical goal in this phase shifts to promoting wound healing, preventing infection recurrence, and optimizing metabolic control. The healing time of DFU wounds can range from several days to several months. Although this relatively stable phase presents an opportunity for GLP-1 RA application, a cautious approach remains essential. We recommend starting with the lowest effective dose, preferring daily formulations, and gradually transitioning to weekly formulations while adjusting the dosage or discontinuing the drug based on gastrointestinal reactions. The benefits of GLP-1 RA in this phase appear to include its glucose-lowering effect, which may improve wound healing under hyperglycemic conditions, its weight-reducing effect alleviates pressure on the foot, and its cardiovascular protective effect lays a foundation for the patient’s long-term prognosis. Nevertheless, further clinical studies are required to fully elucidate the benefits in this stage.

The prevention phase represents the optimal stage in the natural course of DFU. Foot ulcers have completely healed, skin integrity has been restored, but patients still face a high risk of recurrence and death. During this phase, the patient is generally in good health, with no acute infections, and normal oral intake. This phase may present the most appropriate time for GLP-1 RA application, but its long-term benefits should be carefully weighed against individual patient needs and potential risks. Long-term use of GLP-1 RA could potentially achieve several therapeutic goals: strict glucose control slows the progression of DPN, weight reduction decreases foot pressure, vascular function improvement enhances lower limb blood flow, cardiovascular protection reduces the main mortality risk in DFU patients, and renal protection improves DFU-related renal function deterioration. However, careful monitoring is advised, and selecting the appropriate drug type based on the patient’s specific condition to maximize its positive impact on the long-term prognosis of DFU patients. **Table 1** compares the applicability of GLP-1 RA in DFU.

**Table 1:** Comparison of the applicability of GLP-1 RA in DFU.

Drug type	Monotarget	Dual-target	Triple/multitarget
Main targets	GLP-1R	GLP-1R/GIPR or GLP-1R/GCGR	GLP-1R/GIPR/GCGR (or other target)
Representative drug	liraglutide, semaglutide	tirzepatide, mazdutide	retatrutide
Metabolic benefits	good weight loss, blood sugar control	better weight loss, blood sugar control	expected optimal weight loss, blood sugar control
Cardiovascular benefits	some drugs reduce the risk of MACE (8–10)	underway or planned CVOT	underway or planned CVOT
Liver fat improvement	effective	significant, reduction in liver fat content can reach up to 50% (26)	excellent, reduction in liver fat content can reach 86% (31)
Main adverse reactions	gastrointestinal discomfort (nausea, vomiting, diarrhea)		
Main monitoring indicators	dietary intake, albumin, hemoglobin, muscle mass, heart rate		
Recommend DFU population	wound healing period/prevention period, high compliance	wound healing period/prevention period, need cautious evaluation	prevention period, need strict patient selection and cautious initiation

DFU, diabetic foot ulcer; GLP-1R, glucagon-like peptide-1 receptor; GIPR, glucose-dependent insulinotropic polypeptide receptor; GCGR, glucagon receptor; MACE, major adverse cardiovascular event; CVOT, cardiovascular outcome trial.

## 6 Conclusion and clinical implications

The high mortality rate of DFU essentially reflects its systemic vascular disease characteristics, rather than solely local foot lesions. The advent of GLP-1 RA provides new hope for treating this complex disease, with its cardiovascular protective effects confirmed in CVOTs offering a powerful tool for improving the long-term prognosis of DFU patients. However, the success of this drug’s application depends on clinicians’ accurate judgment of the patient’s disease stage and the corresponding refined treatment decisions.

In the acute infection phase of DFU, GLP-1 RA may do more harm than good and should be avoided or suspended. In the subacute recovery phase, cautious initiation, slow dosage increase, and close monitoring are needed. In the chronic prevention phase, it should be considered as one of the effective cardiovascular protection strategies. In the future, with the clinical application of dual-target, triple-target, and even multitarget GLP-1 RA, stronger and more comprehensive metabolic improvements may be provided to DFU patients, potentially lowering the mortality risk of DFU. However, the realization of this prospect requires clinical trial validation for specific DFU patient populations and continuous clinical observation and decision-making enhancement by clinicians.

## References

[1] Armstrong DG Boulton AJM Bus SA . Diabetic foot ulcers and their recurrence. N Engl J Med. (2017) 376:2367–75. doi: 10.1056/NEJMra1615439, PMID: 28614678

[2] Schaper NC van Netten JJ Apelqvist J Bus SA Fitridge R Game F . Practical guidelines on the prevention and management of diabetes-related foot disease (IWGDF 2023 update). Diabetes Metab Res Rev. (2024) 40:e3657. doi: 10.1002/dmrr.3657, PMID: 37243927

[3] Chen L Sun S Gao Y Ran X . Global mortality of diabetic foot ulcer: A systematic review and meta-analysis of observational studies. Diabetes Obes Metab. (2023) 25:36–45. doi: 10.1111/dom.14840, PMID: 36054820

[4] Armstrong DG Tan TW Boulton AJM Bus SA . Diabetic foot ulcers: A review. JAMA. (2023) 330:62. doi: 10.1001/jama.2023.10578, PMID: 37395769 PMC10723802

[5] Chin BZ Lee P Sia CH Hong CC . Diabetic foot ulcer is associated with cardiovascular-related mortality and morbidity – a systematic review and meta-analysis of 8062 patients. Endocrine. (2024) 84:852–63. doi: 10.1007/s12020-024-03696-5, PMID: 38280983

[6] Highton P Abdala R Evley R Balasubramanian V Davies M Dhatariya K . The use of SGLT2 inhibitors in people with diabetes-related foot disease: A Delphi-based consensus study. Diabetes Obes Metab. (2025) 27:4537–46. doi: 10.1111/dom.16498, PMID: 40495570 PMC12232341

[7] Hong AT Luu IY Lin F Shin L Hsu C-H Shih C-D . Differential effect of GLP-1 receptor agonists and SGLT2 inhibitors on lower-extremity amputation outcomes in type 2 diabetes: A nationwide retrospective cohort study. Diabetes Care. (2025) 48:1728–36. doi: 10.2337/dc25-0292, PMID: 40560644 PMC12451831

[8] Marso SP Daniels GH Brown-Frandsen K Kristensen P Mann JFE Nauck MA . Liraglutide and cardiovascular outcomes in type 2 diabetes. N Engl J Med. (2016) 375:311–22. doi: 10.1056/NEJMoa1603827, PMID: 27295427 PMC4985288

[9] Gerstein HC Colhoun HM Dagenais GR Diaz R Lakshmanan M Pais P . Dulaglutide and cardiovascular outcomes in type 2 diabetes (REWIND): a double-blind, randomised placebo-controlled trial. Lancet. (2019) 394:121–30. doi: 10.1016/S0140-6736(19)31149-3, PMID: 31189511

[10] Marso SP Bain SC Consoli A Eliaschewitz FG Jódar E Leiter LA . Semaglutide and cardiovascular outcomes in patients with type 2 diabetes. N Engl J Med. (2016) 375:1834–44. doi: 10.1056/NEJMoa1607141, PMID: 27633186

[11] Drucker DJ . Mechanisms of action and therapeutic application of glucagon-like peptide-1. Cell Metab. (2018) 27:740–56. doi: 10.1016/j.cmet.2018.03.001, PMID: 29617641

[12] Zhu Y Li Y Ma Y Zhang X Du X Gao J . Liraglutide accelerates ischemia-induced angiogenesis in a murine diabetic model. J Am Heart Assoc. (2023) 12:e026586. doi: 10.1161/JAHA.122.026586, PMID: 36789853 PMC10111486

[13] Moustafa PE Abdelkader NF El Awdan SA El-Shabrawy OA Zaki HF . Liraglutide ameliorated peripheral neuropathy in diabetic rats: Involvement of oxidative stress, inflammation and extracellular matrix remodeling. J Neurochem. (2018) 146:173–85. doi: 10.1111/jnc.14336, PMID: 29572844

[14] Ma X Liu Z Ilyas I Little PJ Kamato D Sahebka A . GLP-1 receptor agonists (GLP-1RAs): cardiovascular actions and therapeutic potential. Int J Biol Sci. (2021) 17:2050–68. doi: 10.7150/ijbs.59965, PMID: 34131405 PMC8193264

[15] Huang H Wang L Qian F Chen X Zhu H Yang M . Liraglutide via activation of AMP-activated protein kinase-hypoxia inducible factor-1α-heme oxygenase-1 signaling promotes wound healing by preventing endothelial dysfunction in diabetic mice. Front Physiol. (2021) 12:660263. doi: 10.3389/fphys.2021.660263, PMID: 34483951 PMC8415222

[16] Bechara N Gunton JE Flood V Hng TM McGloin C . Associations between nutrients and foot ulceration in diabetes: A systematic review. Nutrients. (2021) 13:2576. doi: 10.3390/nu13082576, PMID: 34444735 PMC8400510

[17] Basiri R Spicer M Munoz J Arjmandi B . Nutritional intervention improves the dietary intake of essential micronutrients in patients with diabetic foot ulcers. Curr Dev Nutr. (2020) 4:nzaa040_008. doi: 10.1093/cdn/nzaa040_008

[18] Bellavance D Chua S Mashimo H . Gastrointestinal motility effects of GLP-1 receptor agonists. Curr Gastroenterol Rep. (2025) 27:49. doi: 10.1007/s11894-025-00995-3, PMID: 40622491

[19] Gorgojo-Martínez JJ Mezquita-Raya P Carretero-Gómez J Castro A Cebrián-Cuenca A De Torres-Sánchez A . Clinical recommendations to manage gastrointestinal adverse events in patients treated with glp-1 receptor agonists: A multidisciplinary expert consensus. J Clin Med. (2022) 12:145. doi: 10.3390/jcm12010145, PMID: 36614945 PMC9821052

[20] Drucker DJ . The biology of incretin hormones. Cell Metab. (2006) 3:153–65. doi: 10.1016/j.cmet.2006.01.004, PMID: 16517403

[21] Lewis JE Omenge DK Patterson AR Anwaegbu O Tabukum NN Lewis JE . The impact of semaglutide on wound healing in diabetes related foot ulcer patients: A TriNetX database study. Diabetes Vasc Dis Res. (2025) 22:14791641251322909. doi: 10.1177/14791641251322909, PMID: 40080656 PMC11907515

[22] Holst JJ Rosenkilde MM . GIP as a therapeutic target in diabetes and obesity: insight from incretin co-agonists. J Clin Endocrinol Metab. (2020) 105:e2710–6. doi: 10.1210/clinem/dgaa327, PMID: 32459834 PMC7308078

[23] Dutta D Surana V Singla R Aggarwal S Sharma M . Efficacy and safety of novel twincretin tirzepatide a dual GIP and GLP-1 receptor agonist in the management of type-2 diabetes: A Cochrane meta-analysis. Indian J Endocrinol Metab. (2021) 25:475–89. doi: 10.4103/ijem.ijem_423_21, PMID: 35355921 PMC8959203

[24] Novikoff A Müller TD . The molecular pharmacology of glucagon agonists in diabetes and obesity. Peptides. (2023) 165:171003. doi: 10.1016/j.peptides.2023.171003, PMID: 36997003 PMC10265134

[25] Ji L Jiang H Li H Tian J Liu D Zhao Y . 1856-LB: efficacy and safety of mazdutide in chinese participants with overweight or obesity (GLORY-1). Diabetes. (2024) 73:1856–LB. doi: 10.2337/db24-1856-LB

[26] Browne SK Suschak JJ Tomah S Gutierrez JA Yang J Georges B . Safety and efficacy of 24 weeks of pemvidutide in metabolic dysfunction-associated steatotic liver disease: A randomized, controlled clinical trial. JHEP Rep. (2025) 7:101483. doi: 10.1016/j.jhepr.2025.101483, PMID: 41113119 PMC12529369

[27] NCT06077864 Study Group . Study details | NCT06077864 | A study to test the effect of survodutide (BI 456906) on cardiovascular safety in people with overweight or obesity (SYNCHRONIZE™ - CVOT). ClinicalTrials.gov. Available online at: https://clinicaltrials.gov/study/NCT06077864 (Accessed November 9, 2025).

[28] Jastreboff AM Kaplan LM Frías JP Wu Q Du Y Gurbuz S . Triple–hormone-receptor agonist retatrutide for obesity — A phase 2 trial. N Engl J Med. (2023) 389:514–26. doi: 10.1056/NEJMoa2301972, PMID: 37366315

[29] Rosenstock J Frias J Jastreboff AM Du Y Lou J Gurbuz S . Retatrutide, a GIP, GLP-1 and glucagon receptor agonist, for people with type 2 diabetes: a randomised, double-blind, placebo and active-controlled, parallel-group, phase 2 trial conducted in the USA. Lancet Lond Engl. (2023) 402:529–44. doi: 10.1016/S0140-6736(23)01053-X, PMID: 37385280

[30] Coskun T Wu Q Schloot NC Haupt A Milicevic Z Khouli C . Effects of retatrutide on body composition in people with type 2 diabetes: a substudy of a phase 2, double-blind, parallel-group, placebo-controlled, randomised trial. Lancet Diabetes Endocrinol. (2025) 13:674–84. doi: 10.1016/S2213-8587(25)00092-0, PMID: 40609566

[31] Sanyal AJ Kaplan LM Frias JP Brouwers B Wu Q Thomas MK . Triple hormone receptor agonist retatrutide for metabolic dysfunction-associated steatotic liver disease: a randomized phase 2a trial. Nat Med. (2024) 30:2037–48. doi: 10.1038/s41591-024-03018-2, PMID: 38858523 PMC11271400

[32] Kharitonenkov A Shiyanova TL Koester A Ford AM Micanovic R Galbreath EJ . FGF-21 as a novel metabolic regulator. J Clin Invest. (2005) 115:1627–35. doi: 10.1172/JCI23606, PMID: 15902306 PMC1088017

[33] Xiao Y Liu L Xu A Zhou P Long Z Tu Y . Serum fibroblast growth factor 21 levels are related to subclinical atherosclerosis in patients with type 2 diabetes. Cardiovasc Diabetol. (2015) 14:72. doi: 10.1186/s12933-015-0229-9, PMID: 26047614 PMC4475300

[34] Zhang X Hu Y Zeng H Li L Zhao J Zhao J . Serum fibroblast growth factor 21 levels is associated with lower extremity atherosclerotic disease in Chinese female diabetic patients. Cardiovasc Diabetol. (2015) 14:32. doi: 10.1186/s12933-015-0190-7, PMID: 25850006 PMC4359481

[35] Liskiewicz D . An unimolecular GLP-1R/GIPR/Pan-PPAR quintuple agonist eliminates obesity and insulin resistance in mice. Available online at: https://www.easd.org/media-centre/!events/25/programs/2025-09-15 (Accessed November 10, 2025).

[36] Liu L Chen J Wang L Chen C Chen L . Association between different GLP-1 receptor agonists and gastrointestinal adverse reactions: A real-world disproportionality study based on FDA adverse event reporting system database. Front Endocrinol. (2022) 13:1043789. doi: 10.3389/fendo.2022.1043789, PMID: 36568085 PMC9770009

[37] Lenharo M . Anti-obesity drugs’ side effects: what we know so far. Nature. (2023) 622:682–2. doi: 10.1038/d41586-023-03183-3, PMID: 37833484

[38] Lubberding AF Veedfald S Achter JS Nissen SD Soattin L Sorrentino A . Glucagon-like peptide-1 increases heart rate by a direct action on the sinus node. Cardiovasc Res. (2024) 120:1427–41. doi: 10.1093/cvr/cvae120, PMID: 38832935 PMC11472427

